# Opioid Use and Gut Dysbiosis in Cancer Pain Patients

**DOI:** 10.3390/ijms25147999

**Published:** 2024-07-22

**Authors:** Flaminia Coluzzi, Maria Sole Scerpa, Chiara Loffredo, Marina Borro, Joseph V. Pergolizzi, Jo Ann LeQuang, Elisa Alessandri, Maurizio Simmaco, Monica Rocco

**Affiliations:** 1 Department of Medical-Surgical Sciences and Translational Medicine, Sapienza University of Rome, 00189 Rome, Italy; 2 Unit of Anaesthesia, Intensive Care, and Pain Medicine, Sant’Andrea University Hospital, 00189 Rome, Italy; 3 Department of Neuroscience, Mental Health and Sense Organs NESMOS, Sapienza University of Rome, 00185 Rome, Italy; 4 NEMA Research, Inc., Naples, FL 34108, USA

**Keywords:** opioids, gut dysbiosis, constipation, microbiota, gut–brain axis, tolerance, analgesia, neuroinflammation, PAMORAs

## Abstract

Opioids are commonly used for the management of severe chronic cancer pain. Their well-known pharmacological effects on the gastrointestinal system, particularly opioid-induced constipation (OIC), are the most common limiting factors in the optimization of analgesia, and have led to the wide use of laxatives and/or peripherally acting mu-opioid receptor antagonists (PAMORAs). A growing interest has been recently recorded in the possible effects of opioid treatment on the gut microbiota. Preclinical and clinical data, as presented in this review, showed that alterations of the gut microbiota play a role in modulating opioid-mediated analgesia and tolerability, including constipation. Moreover, due to the bidirectional crosstalk between gut bacteria and the central nervous system, gut dysbiosis may be crucial in modulating opioid reward and addictive behavior. The microbiota may also modulate pain regulation and tolerance, by activating microglial cells and inducing the release of inflammatory cytokines and chemokines, which sustain neuroinflammation. In the subset of cancer patients, the clinical meaning of opioid-induced gut dysbiosis, particularly its possible interference with the efficacy of chemotherapy and immunotherapy, is still unclear. Gut dysbiosis could be a new target for treatment in cancer patients. Restoring the physiological amount of specific gut bacteria may represent a promising therapeutic option for managing gastrointestinal symptoms and optimizing analgesia for cancer patients using opioids.

## 1. Introduction

Opioids are still a cornerstone in the management of severe chronic pain, particularly in cancer patients [1]. However, their use is burdened by a number of side effects, particularly those involving the gastrointestinal system, which include xerostomia, nausea, vomiting, gastro-esophageal reflux, abdominal bloating, abdominal pain and cramping, anorexia, oral malodor, a sense of incomplete evacuation, and constipation. This complex clinical picture, named opioid-induced bowel dysfunction (OIBD), has a great impact on patients’ quality of life and adherence to opioid therapy [2,3] and finds its underlying cause in the activation of mu-opioid receptors (MOR) in the enteric nervous system, namely the myenteric and the submucosal plexus, as well as delta-opioid receptor (DOR) activation, the latter probably leading to an inhibition of secretomotor neurons and consequently reduced water and chloride ions passing into the intestinal lumen [4]. Furthermore, epigenetic alterations were found to correlate with possibility of adverse effects, namely dry mouth and constipation, suggesting that opioid-induced DNA methylation of certain genes may lead to OIBD, among other adverse events [5].

Atypical opioids, namely tramadol [6] and tapentadol [7], are known to have a reduced “mu-load”, hence they may display a better safety profile with regard to enteric adverse effects. Particularly, opioid-induced constipation (OIC) is still one of the main limitations to optimizing analgesia in opioid users, because, conversely to other adverse effects, tolerance does not build up and OIC may persist throughout the duration of treatment [8,9]. In fact, prolonged exposure to opioids generally leads to the activation of β-arrestin2 in the CNS with consequent tolerance development, whilst this protein is downregulated in the ileum. Moreover, specific intracellular pathways, receptor internalizations and recycling patterns, and receptor variants may be activated in the colon after prolonged opioid consumption [10]. In order to reverse OIC, pharmacological research in the last few years has mainly focused on the discovery of peripherally acting mu-opioid receptor antagonists (PAMORAs), namely methylnaltrexone, naloxegol, and naldemedine, which exert their antagonist activity on gastrointestinal MORs without impairing opioid-induced analgesia since they cannot cross the blood–brain barrier [11]. Furthermore, naldemedine was found to reach brain regions famously implied with nausea and vomiting, since these areas are not protected by the BBB; hence, naldemedine may have a role in controlling opioid-induced nausea and vomiting, which is a burdensome feature of OIBD [4], and may be even more so in cancer patients already suffering from it because of cancer treatments. Furthermore, a growing interest has been noted in the possible effects of opioid treatment on the gut microbiota, alongside the risks connected with an altered microbiota pattern in patients suffering from cancer pain [12]. Gut dysbiosis could probably contribute to the gastro-intestinal symptoms observed in OIBD; on the other hand, further implication of gut dysbiosis in opioid-related effects, as in reward and addiction processes, is still under debate [13], as is the possible role of PAMORAs in reversing opioid-induced dysbiosis [10].

The aim of this review is to analyze the relationship between opioid use and gut dysbiosis, and its potential clinical consequences in cancer patients. 

## 2. Gut Microbiota: Definitions, Basic Concepts, and Methodologies

The mixture of commensal, symbiotic, and pathogenic microorganisms living in a specific part of the human body represents a community with its own peculiar metabolic homeostasis, determined by specific microenvironmental features shaped by the microbial–host crosstalk. Homeostasis disruption (dysbiosis) with qualitative and quantitative alteration in the microflora composition may precede or be consequent to pathologies [14,15]. 

Knowledge about “microbiota” or “microbiome” functions in human health and disease dramatically increased in the last decade thanks to Next-Generation Sequencing (NGS) technologies, allowing for rapid and massive DNA analysis. The term microbiota usually refers to the composition of each microbial community, i.e., to the type and relative abundance of microbes living in a specific niche, while the term “microbiome” (or “metagenome”) usually refers to the whole (collective) genomic makeup of that community, including plasmids [16]. Although both concepts theoretically include the analysis of bacteria, fungi, and viruses, microbiota studies usually just deal with bacteria. 

NGS analysis is based on the simultaneous characterization of DNA from all microbes coexisting in the same, complex biological sample. The obtained sequences are clustered and compared with microbial DNA reference databases: the level of similarity with evolutionarily conserved, taxon-specific sequences allows for microbe identification. Microbiota analysis is commonly performed by NGS of a single bacterial gene, encoding 16S ribosomal RNA. This gene is highly conserved, but is spanned by nine hypervariable regions, termed V1–V9, which are more taxon-specific [17]. Thus, the information derived from 16S analysis is limited to the type and relative abundance of the bacteria residing in the biological sample. Albeit limited, this approach is rapid and cheap, and thus widely used. In contrast, studying the microbiome requires whole-genome sequencing of all the microorganisms present in the analyzed samples, giving information on the metabolic capability of the population, as well as identifying microbes, including fungi and viruses. Thus, the concept of dysbiosis explored by metagenomics is wider, covering the functional assessment of the microbial community. The approach is more laborious and expensive and requires complex statistical and bioinformatic pipelines, which presently limit its application. 

The human gut microbiome represents a major field of research, and a bulk of knowledge has been accrued about its development, functions, and modifying factors. The establishment of a peculiar core microflora in the human gut is determined at birth, when vaginal delivery or cesarean section expose the infant to a different profile of first colonizing bacteria, which then shape the evolution of the infant’s microbial community. Also, breast- or formula-feeding affect the composition of the gut microbiota, which in adulthood will reach a definite and highly personal composition based on both host-related and external factors, such as diet [18,19,20]. 

The plethora of functions exerted by the gut microbiota explains its implication in so many human pathologies [21,22] and can be roughly summarized as, (i) metabolic: gut microflora expresses a huge number of genes compared to the host, allowing additional biochemical reactions which contribute to energy extraction from food, vitamin production, and bile biotransformation [23]; (ii) protective: besides contributing to the functional efficiency of the gastro-intestinal barrier and limiting the proliferation of pathogenic microbes by nutrient competition and the secretion of antimicrobial substances, the role of the gut microbiota in the development and homeostasis of the intestinal mucosal immune system is described well [24]; (iii) neurological: the gut microbiota is involved in the gut–brain axis at both local and distant levels [25]. 

Despite the great expectation concerning novel treatment targets offered by the microbiota, it should be kept in mind that the systematization and methodological standardization of knowledge are presently an unresolved pitfall of microbiota/microbiome research [26,27], representing the main limit to a broad clinical application of specific diagnostic and intervention tools, aiming to recognize and treat gut dysbiosis.

## 3. The Effects of Opioid Use on the Gut Microbiota

Recently, plentiful literature has arisen about the effects of opioids on the gut microbiota, starting from the evidence that the most common side effects of these drugs are reduced GI motility and subsequently severe constipation. Thus, it is reasonable to assume that opioids could have an impact on the microbiota itself. The majority of the studies on this topic were performed on animal models, given the complexity of human models. Moreover, the variety of dietary patterns and drug use may be a confounding factor for the phylogenetic and metabolic analyses needed to examine the interaction between the gut and brain, and their consequences on host physiology. 

### 3.1. Preclinical Evidence

Most experimental studies, performed in mice, reported dysbiosis as a consequence of opioid treatment, although specific alterations in microbial species vary among studies. Wang et al. [28] and Lee et al. [29] found an increase in fecal specimens of *Flavobacterium*, *Enterococcus*, *Fusobacterium*, *Sutterella*, *Clostridium*, *Firmicutes*, and *Ruminococcus* species, isolated from mice treated with a subcutaneous morphine implant or intraperitoneal injections. On the other hand, Kang et al. reported a significant reduction in *Bacteroidetes* and *Firmicutes* and an increase in *Proteobacterias (Enterobacteriales)* in the fecal samples of mice, five days after morphine pellet implantation [30]. In these animal studies, changes in the microbiome pattern have been detected since the first days of treatment [28], suggesting a possible effect not only after chronic exposure, but also in acute pain management with MOR agonists. 

Chronic morphine exposure even resulted in disorganization of the tight junctions in the colon, leading to a disruption of the epithelial integrity, enhanced permeability, bacterial translocation, and subsequent chronic inflammation [30]. Banerjee et al. demonstrated that morphine may induce global changes in the gut microbiota, compromise the gut barrier, and disrupt cholesterol/bile acid metabolism. They reported an increase in *Firmicutes* after morphine exposure in mice, therefore reducing the *Bacteroidetes/Firmicutes* ratio in those animals [31]. Similar changes have been observed in chronic conditions characterized by systemic inflammation, such as obesity and aging [32]. Chronic morphine and fentanyl exposure has also been associated with impairment of the antimicrobial activity of the intestinal epithelium, which may be restored by oral supplementation of butyrate [33].

Chronic opioid use is widely known to be associated with addiction, tolerance, and hyperalgesia [8]. Recent evidence suggests a possible role for dysbiosis in these processes. Zhang et al. found a selective depletion in both *Lactobacillaeae* and *Bifidobacteria* in morphine-tolerant mice, alongside a disruption in gut integrity, facilitated bacterial translocation, and over-expression of TLR2 and TLR4, which are the major receptors mediating the host’s response to Gram-positive and Gram-negative bacteria, respectively [34]. Particularly, TLR2 seems to be mostly associated with opioid tolerance in animal studies, suggesting a particular role of Gram-positive bacteria, namely Enterococcus, in this process. Interestingly, morphine-induced dysbiosis supported local gut inflammation and maintenance of opioid tolerance; however, both antibiotic [35] and probiotic treatments were shown to be effective in attenuating analgesic tolerance and improving morphine efficacy [34]. Although the precise mechanism by which morphine may induce epithelial disruption remains unknown, a role of µ-opioid receptors (MOR) and toll-like receptor (TLR) signaling has been proposed [36,37]: in murine models for colitis, MOR activation led to the activation of inflammatory responses through an increased migration of immune cells and TLR-mediated disruption of tight junctions [37].

Alterations of the gut microbiota are of utmost importance in chronic opioid users, since they play a role in developing severe constipation, which is currently considered as the most important side effect of chronic opioid use, as a consequence of the activity of exogenous opioids on MOR dislocated in the myoenteric and submucosal plexuses. According to several studies, while severe opioid-induced constipation is associated with barrier disruption and bacterial translocation, which enhance systemic inflammatory response overall [38], non-opioid-induced constipation (for instance, resulting from low food intake or a low-fiber diet) is not associated with alterations of the epithelium and microbiota translocation. This observation highlights the different underlying mechanism that supports different types of constipation [31].

Nonetheless, opioid administration was found to alter microbial composition even after brief exposures in murine models, and such modifications not only endured in the few days after morphine treatment [39], but were also linked to dysbiosis in offspring after opioids, namely hydromorphone, were administrated in pregnant mice [40]. Accordingly, after a 2-week period of prenatal hydromorphone administration, followed by methadone exposure as a model for maintenance treatment, Abu et al. found altered microbiome and enhanced sensitivity to mechanical and thermal pain in mice offspring, which were reverted via supplementation of the probiotic VSL#3 [41] (Table 1).

### 3.2. Clinical Evidence

Literature is currently poor in terms of clinical studies, for different reasons. The complexity of human models and the numerous confounding factors that can alter microbiota composition, such as diet, drug exposure, and comorbidities, make clinical studies hard to conduct and results challenging to interpret. Moreover, it is still unclear whether preclinical studies could be transposed to clinical use, because the human gut microbiota is quite different from those of mice and rats, and more similar to Non-Human Primates (NHPs), such as monkeys. The human microbiota is dominated by *Bacteroides* followed by *Ruminococcaceae* and *Clostridiales*. Rats and NHPs show a higher prevalence of *Prevotella*, while mice present members of the *family S24-7* and *Clostridiales*. These host species-specific gut microbiota signatures may reflect disparities in host factors. Unfortunately, studies using NHP models are limited [42]. However, Sindberg et al. first found that morphine administration in NHPs changes metabolite profiles and bacterial composition, with a decrease in *Streptococcaceae streptococcus* and *Pasteurellaceae Aggregatibacter*, especially exacerbating simian immunodeficiency virus (SIV)-mediated dysbiosis in the early stages of infection [43].

Dysbiosis itself may be defined as a “microbiota community associated with a diseased state that can be differentiated from the microbiota community associated with a healthy control state” [44]. Even though it is well known that the microbiota is essential for gut health, its exact role in maintaining this homeostasis is still unclear [45].

Chronic opioid use has been reported as an independent factor for increased hospital readmission in cirrhotic patients, regardless of hepatic encephalopathy (HE). In these patients, opioid-induced constipation may worsen bowel overgrowth and bacterial translocation. Moreover, they were diagnosed with a reduction in *Bacteroidaceae* and bacterial metabolic products and an increase in endotoxin and interleukin-6 (IL-6) levels [46]. Opioid administration also correlated with increased *Bifidobacterium* presence in diabetic African American men, with a significant interaction between opioid use, type 2 diabetes, and metformin administration, particularly on *Bifidobacterium* and *Prevotella* abundance [47].

## 4. The Gut Microbiota–Brain Axis

**Figure 1:**
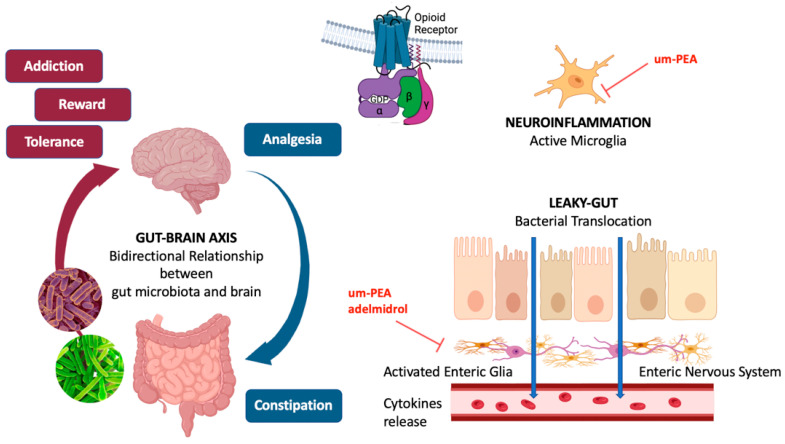
Mechanisms of opioid-mediated modifications of the gut microbiota. Opioids ensure analgesia and cause constipation through their activity on mu-opioid receptors respectively in the central and enteric nervous system. Leaky-gut has been recognized as the main mechanism of bacterial translocation, which activates enteric glia, leading to the massive release of pro-inflammatory mediators. The resulting altered gut microbiota has been implicated in most of the challenging conditions related to chronic opioid use, such as tolerance, addiction, and reward. The bidirectional relationship between the gut microbiota and the brain play a key role in the well-known gut–brain axis.

Nearly 80% of microbes of the human body reside in the gut. This rich microbiome is believed to somehow “communicate” with other systems and apparatuses. The gut microbiota–brain axis has recently been identified as an entity encompassing the microbiome hosted in the gut and the brain. Currently, the precise mechanism of communication between these two entities is not fully understood, but there is evidence that a bidirectional interaction may exist (Figure 1). On one hand, the gut microbiota can influence the structure and functionality of the central nervous system (CNS), hence modulating behavior and cognitive development. For example, the administration of probiotics had beneficial effects in animal models on both depression and autism spectrum disorder (ASD). On the other hand, the central nervous system regulates several functions of the GI tract and the ENS, namely motility, acid, bicarbonates, and mucus production and secretion, fluid balance through the epithelium, immunological response, and so on [28,48].

Several possible ways of communication between the CNS have been identified: (i) through the sympathetic and parasympathetic branches of the autonomic nervous system (ANS), (ii) the hypothalamic–pituitary–adrenal (HPA) axis, (iii) the gut immune system, a humoral communication through neurotransmitters and molecules synthetized by gut bacteria; (iv) the gut–mucosal barrier and (v) the blood–brain barrier (BBB) [49]. Bacteria in the gut can produce and/or consume several known neurotransmitters, for instance gamma-aminobutyric acid (GABA), norepinephrine, dopamine, and serotonin. Modifications in the gut microbiome may be responsible for altered levels of neurotransmitters in the gut, blood, and the CNS, hence causing neurological disorders [50], especially through epigenetic mechanisms, including DNA acetylation and methylation, promoted by bacteria-derived metabolites. Consequently, supplementation with pre- or probiotics was shown to ameliorate neurobehavioral pathological patterns [51].

## 5. Gut Dysbiosis and Neuroinflammation 

Alterations in the gut microbiome have been widely associated with many pathological conditions of the nervous system, such as depression [52], anxiety [53], autism spectrum disorders (ASD) [54,55], schizophrenia [56], multiple sclerosis [57], Parkinson’s disease [58], Alzheimer’s disease [59], bipolar disorder [60], and substance use disorder (SUD) [61].

Prolonged morphine administration causes the so-called “leaky-gut”, characterized by a disrupted intestinal epithelial barrier, which permits bacterial translocation [36]. Morphine-mediated activation of TLRs in epithelial cells cause the transfer in the bloodstream of pathogen-associated molecular patterns (PAMPs), like lipopolysaccharide (LPS), lipoteichoic acid (LTA), peptidoglycan (PGN), and beta-glucan, which activate immune cells and enteric glial cells [62,63]. Enteric glia is mainly located in the myenteric and submucosal plexuses of the enteric nervous system and is involved in the process of gut barrier disruption. Despite not being completely understood, growing evidence supports the key role of the enteric glial cells in regulating GI and immune function, through interactions with intestinal neurons. Glial activation by bacterial PAMPs, such as LPS, determine a sustained release of cytokines during morphine therapy [64]. The precise relationship between opioid-induced bacterial translocation, the activation of immune and enteric glial cells, and the development of analgesic tolerance is still unclear, and may rely on both central and peripheral neuroinflammation [35]. However, preclinical studies showed that the development of morphine tolerance was associated with a depletion of specific bacterial communities and may be attenuated through treatment with probiotics [34].

The microbiota may modulate pain regulation and tolerance through peripheral and central mechanisms. In mice chronically treated with morphine, loss of MOR in the dorsal root ganglia (DRG) neurons completely abrogates analgesic tolerance, defined as a gradual decrease of analgesic efficacy at fixed doses [35]. Accordingly to these findings, in mice chronically treated with morphine, loss of MOR in the DRG neurons completely abrogates analgesic tolerance. The same results have been observed for opioid-induced hyperalgesia (OIH), described as increased pain after normally noxious stimuli [65] and for pro-nociceptive long-term potentiation, which describes a modification in synaptic plasticity [66], probably accountable for both tolerance and OIH [67]. 

Gut bacteria-derived PAMPs, released into the bloodstream, activate immune cells through TLRs and provoke cytokine and chemokine release, thus eliciting systemic inflammation and indirect sensitization of primary sensory neurons in DRGs [68]. PAMPs can also directly activate primary sensitive neurons by binding to specific receptors; for example, LPS can bind to TLR4 and induce the sensitization and activation of nociceptive neurons in DRGs [69]. Short chain fatty acids (SCFAs) contribute to gut microbiota-related pain modulation via multiple mechanisms, mainly acting on FFAR2-3 and regulating leucocyte activation and the production of cytokines (TNF-α, IL-6, IL-2, and IL-10), chemokines, and eicosanoids [70]. Butyrate can decrease pain sensation and TNF-α levels in experimental models [71] and its administration in patients suffering from inflammatory bowel disease (IBD) abdominal pain can relieve pain sensation [72].

Recent evidence has also highlighted the role of GI bacteria in promoting the development, maturation, and function of microglia in the CNS [73]. Once activated, microglial cells release inflammatory cytokines and chemokines and sustain an increased excitatory glutamatergic neurotransmission and a decreased GABAergic tone, leading to central sensitization and hyperalgesia [74]. These phenomena, known as “neuroinflammation”, play a key role in most chronic pain syndromes [75], as well as several stress-related states. Interestingly, gut bacteria may not be the only microorganisms in the microbiome responsible for such responses: in fact, alterations in the gut virome were recently linked to the development of stress-associated behavioral patterns through the activation of pro-inflammatory cells and cytokine release, alterations of gut bacteriome, and even altered gene expression in the CNS [76].

Palmitoylethanolamide (PEA), an endogenous lipid mediator belonging to the N-acylethanolamine (NAE) family, plays a local autacoid role in controlling inflammation and in analgesic phenomena [77]. Recent studies have supported the hypothesis that ultramicronized-PEA (um-PEA) administration counteracts neuronal alterations, reduces morphine tolerance [78], and potentiates morphine analgesia without increasing the morphine’s doses over time [79]. Um-PEA ranges from 0.8 to 6 μm, which is the size that ensure oral absorption and optimal distribution to the central nervous system. Um-PEA delays the development of tramadol tolerance, potentiating either oxycodone or tramadol analgesia and allowing a long-lasting analgesic effect with a low-dose regimen of both opioids [80]. These interesting pieces of evidence on the role played by um-PEA on the delay of opioid tolerance and on the enhancement of opioids’ analgesic effects leads to the hypothesis that um-PEA may restore gut microbiota homeostasis, which is altered in chronic opioid users. Although specific data are not yet available in the literature, it is well known that um-PEA administration to BTBR mice, which are recognized as a valid preclinical model of the core autism symptom domains, and to vitamin D deficient mice, is able to restore gut homeostasis by improving gut integrity, remodeling the fecal microbiota profile, and raising the *Firmicutes/Bacteroidetes* ratio and some specific commensal gut bacteria, such as *Akkermansia muciniphila* [81,82]. 

Similarly, adelmidrol, which is a well-known endogenous PEA enhancer with proven anti-inflammatory properties in different chronic inflammatory conditions [83], has been shown to increase PEA levels in the duodenum and colon [84]. Therefore, its use could maximize the effect of PEA in restoring gut homeostasis.

## 6. Consequences of Gut-Microbiota Alterations on Reward and Addiction Processes

The reward system is made up of a complex interaction of neural structures that regulates many psychological processes, such as “liking” and “wanting”, which are crucial to the reward-behavior circuit. The neurotransmitters involved in these processes are principally dopamine (DA), γ-amino-butyric acid (GABA), and endogenous opioids [85]. 

The reward system can be activated by both “natural” (e.g., sex or food) and “synthetic” stimuli, (such as drugs of abuse and alcohol) and both are processed by meso-cortico-limbic structures, such as the Ventral Tegmental Area (VTA), *nucleus accumbens* (NA), and amygdala. Opioids and other “non-natural” pharmacological reinforcers may induce DA release in these crucial areas, to a greater extent and duration than natural stimuli, leading to addiction. Moreover, repeated exposure to an addictive drug may cause neurophysiological changes, which contribute to the deterioration of addiction [86].

In recent literature, the gut microbiota has been highlighted as a key factor in modulating neurotransmission, particularly in the neural pathways involved in reward, addiction-related actions, stress, and motivation. There is a bidirectional crosstalk between gut bacteria and the central nervous system (CNS) [87,88]. The microbiota has been recognized as a key regulator of the tryptophan metabolism, with a dual effect on serotonin (5-HT) synthesis and kynurenine pathway [89]. Moreover, intestinal bacteria can produce SCFAs under anaerobic conditions, mostly butyrate, propionate and acetate; they are normally used as an energy substrate, but they are also able to activate intracellular signaling by binding to free-fatty acid receptors (FFARs) and cross the blood–brain barrier (BBB) through specific transporters and exert their effects on neuronal and glial cells [90]. SCFAs are able to modulate serotoninergic, GABAergic and dopaminergic neurotransmission in vivo [91] especially in striatum and hippocampus, both crucial areas to reward behavior [92]. The gut microbiota and SCFAs are crucial in modulating morphine reward and exert a key role in morphine addictive behavior [52].

Shishov et al. reported that certain *E. coli* subtypes can produce and degrade monoamines, such as DA, NA, and 5-HT, through specific enzymes [93]. Similarly, *Escherichia coli*, *Lactobacillus*, and *Bifidobacterium* genera have been shown to produce GABA [94]. The vagal gut–brain axis plays a pivotal role in reward and motivation, influencing host response to various rewards, included drugs [95].

Addiction is a chronic disorder characterized by an alteration in motivation, stress, and reward response. It has been demonstrated that both chronic and acute stress play a role in modulating host response to natural and non-natural rewards, and that is a crucial risk factor for developing drug abuse and addiction [96]. A bidirectional relationship has been demonstrated between gut dysbiosis and stress, where imbalances in gut bacteria cause an amplification of the hypothalamic–pituitary–adrenal (HPA) axis stress response, starting a vicious circle [97]. Therefore, gut microbiota imbalance, as caused by opioids, has a role in the development and worsening of addiction to opioids [98] as well as other licit or illicit substances [99]. 

## 7. Opioid-Induced Gut Dysbiosis in Cancer Patients

In the last few years there has been a growing interest in the literature about the role of the gut microbiota in cancer patients. The most investigated topics were, on one hand, the relationship between dysbiosis and carcinogenesis, and on the other hand, the effect of dysbiosis on the effectiveness of cancer treatments. Few data are still available on the more complex relationship between opioid use, the gut microbiota, and cancer treatment. Therefore, in the specific subset of cancer patients, clinicians may raise the question about the interference of opioid-induced gut dysbiosis and the efficacy of chemotherapy and immunotherapy. 

A correlation between gut dysbiosis and carcinogenesis, as well as poor responsiveness to anti-cancer treatments, may be plausible [100]. Modulation of the gut microbiota has been identified as a potential strategy for overcoming resistance to immunotherapy. The intestinal microbiota plays a key role particularly in the response to immune checkpoint inhibitors (ICIs) [101]. Certain specific bacterial compositions such as *Akkermansia*, *Ruminococcaceae*, *Faecalibaterium*, *Bacteroides*, and *Bifidobacterium* have been associated with better outcomes when using ICIs, including a reduction in tumor growth and an increase in prolonged progression-free survival (PFS) [102].

Nowadays, there is no evidence that opioid use may affect the efficacy of chemo- or immunotherapy through gut dysbiosis. Neither do specific opioids seem to increase the risk more than others. However, a retrospective study, conducted on a cohort of 442 metastatic non-small cell lung cancer (NSCLC) patients, showed that antibiotic and opioid administration were related to an overall decreased survival, without any statistically significant difference between the chemotherapy and immunotherapy group. Authors explained these findings as a consequence of confounding factors rather than a real opioid- and antibiotic-induced imbalance in the microbiota [103]. Similarly, a retrospective study in melanoma patients evaluated all potential interactions between drugs known to modify the gut microbiota, included opioids, and overall survival. Only antibiotics were associated with shorter survival in ICI-treated patients [104]. A recent study on 8870 patients treated with ICIs for different types of stage 4 cancer (NSCLC, urothelial carcinoma, and melanoma) revealed that both corticosteroids and opioids, prescribed within 30 days before ICI initiation, strongly correlated with poor prognosis [105].

ICIs work by blocking checkpoint proteins from binding their partner protein, and therefore, by allowing T cells to increase their antitumor activity. Clearly, any medication that may interfere with the immune system is supposed to impair their efficacy. For this reason, the impact of analgesics used for alleviating cancer-related pain on the efficacy of ICIs represent a hot topic in the current literature. Opioids are supposed to repress the immune system through different mechanisms, for example by altering T-cell maturation and function and intestinal microbe composition [98]. Opioids may compromise the immune response by impairing the immune system, may directly act on cancer cells, or may indirectly act on the surface barriers located in the gut. Conversely, COX inhibitors seem to have a favorable effect on the immune system [106], but their use is not suitable for long-term treatment, nor for severe chronic cancer pain.

Clearly, cancer patients with advanced diseases are more likely to use opioids and, at the same time, are more likely than others to develop resistance to chemotherapy and to die. Therefore, it is difficult to correlate opioid use with cancer survival, and specifically with eventual poor outcomes to chemotherapy or immunotherapy. A recent cohort study, conducted on over 1700 patients, showed that long-term opioid use before cancer diagnosis is, by itself, associated with a poor overall outcome [107].

Further research is warranted to discover the potential role of opioids on the gut microbiota and related immuno- and chemotherapy effects. 

Finally, opioid-induced constipation leads to a wide use of laxatives, prokinetics, antispasmodics, and peripherally acting modulators among cancer patients. Osmotic laxatives have been shown to disrupt the gut microbiota and render mice susceptible to *Clostridium difficile* colonization [108].

Despite the fact that the mechanism by which opioids induce constipation is well known, guidelines continue to suggest over-the-counter laxatives as first-line therapy [109]. It is still unclear which laxatives are appropriate to prevent opioid-induced constipation, but the only mechanism-based treatment is the peripheral antagonism of MOR on the enteric nervous system, through PAMORAs [110]. Further research should focus on the possible different effects of traditional laxatives vs. PAMORAs on the maintenance of a healthy gut microbiota.

## 8. Gut Microbiota: A New Target of Treatment 

As above said, alterations in the gut microbiota/microbiome have been implicated in various diseases and inflammatory conditions. Restoring the physiological quantity of specific gut bacteria is now believed to be a valid treatment option. 

### 8.1. Probiotics and Prebiotics

Several studies have highlighted the beneficial effects of probiotics (defined as living microorganisms) and prebiotics (food components able to provide benefits by maintaining a healthy microbiota) [111] on various aspects, ranging from improved digestion and reduced hospitalization rates in cirrhotic patients [112], to ameliorated immune function, and even positive pain modulation. Particularly, *Lactobacillus* was found to induce the expression of mu-opioid receptors in intestinal epithelial cells [113] and in the spinal cord [114]. Preclinical studies have demonstrated a beneficial effect of probiotics in relieving chronic visceral pain. Zhao et al. demonstrated in an IBD animal model that *Clostridium butyricum*, a common gut commensal bacterium, was able to relieve visceral hypersensitivity by reducing bowel inflammation [115]. Similar findings were reported for *Bifidobacterium infantis* [116], *probiotic VSL3* [117], and *Lactobacillus rhamnosus GG* [118] administration in rats. In mice models, VSL3 was also able to attenuate morphine analgesic tolerance [119]. Moreover, a formulation of *Lactobacillus helveticus R0052* and *Bifidobacterium longum R0175* was reported to attenuate the HPA axis-induced stress response [120].

Conflicting findings are available about the effectiveness of probiotics on relieving pain. In a study performed in children, *Lactobacillus reuterii* administration significantly reduced non-surgical abdominal pain [121], while Spiller et al. failed to prove a beneficial effect of *Saccaromyces cerevisiae* administration in improving intestinal pain in patients with IBS [122].

The administration of probiotics has been shown to play a potential role in controlling the adverse effects of cancer therapies, such as oral mucositis and chemotherapy-induced neuropathic pain [123,124]. Moreover, gut microbiota modulation seems to impact the effectiveness and relative outcomes of cancer therapies, such as capecitabine for colorectal cancer pain [125,126].

Prebiotics may also have a role, alone or in combination with probiotics, in chronic pain relief [127]. In a recent study, a mixture of galacto-oligosaccharide was demonstrated to reduce abdominal pain in adults suffering from GI diseases [128]. A combination of specific probiotics with um-PEA could represent an innovative approach for restoring the gut microbiota and preventing opioid-induced disruption of the gut epithelial barrier, by attenuating enteric glia activation.

Future therapies should be oriented toward a “tailored” approach: the identification of specific microbiota alterations may lead to a targeted therapy in order to restore a healthy microbial balance.

### 8.2. Fecal Microbiota Transplantation

Fecal microbiota transplantation (FMT) is a therapy used to treat several diseases, such as *Clostridium difficile* infections, inflammatory bowel disease (IBD), and insulin resistance. It consists of an infusion of liquid filtrated feces from a donor directly into the gut of a patient [129]. Few data are still available on the possible effects of FMT in chronic pain syndromes. Thurm et al. reported a full recovery from pain symptoms in a patient suffering from fibromyalgia after FMT. Interestingly, an increase in *Bifidobacterium* from 0% to 5.23% and a decrease in *Streptococcus* was noticed as related to improved symptoms [130]. Moreover, FMT from naïve donors into chronic morphine treated models can prevent or delay the development of analgesic tolerance [131].

The hypothesized mechanism by which FMT could be helpful in relieving chronic pain is the restoration of a balanced gut microbiota, either directly through competition of pathogenic bacteria or indirectly through stimulation of the intestinal immune system and gut epithelial barrier protection.

## 9. Conclusions

Chronic opioid use leads to several adverse gastrointestinal events, which are not limited to the most commonly known nausea, vomiting, and constipation. Growing evidence in the literature supports an opioid-induced change in gut microbiota, an alteration of the permeability of the epithelial barrier, and an increased risk of bacterial translocation. Unfortunately, most information comes from animal studies, while clinical data on chronic pain patients are currently scarce. With particular regard to cancer patients, who also tend to have an altered food intake, both in quantity and diversity, and are often polymedicated, all of these factors should be taken into consideration, as they may potentially affect their microbiome. Moreover, patients experiencing opioid-induced constipation may further suffer from gut microbiome disturbances due to the misuse/abuse of laxatives, which are currently the first line of treatment. We strongly believe that the early use of PAMORAs, which are the only mechanism-based treatment for OIC, specifically targeting the activation of opioid receptors in the gastroenteric tract, may be a possible ready-to-use solution. Gut dysbiosis is surely a potential target for future constipation research, as it may interfere with the peristaltic action of the intestine. Future investigations should clarify the effects of opioid-induced gut dysbiosis on the gastrointestinal function of opioid users, on chronic pain management, and on the efficacy of anti-cancer therapies.

## Figures and Tables

**Table 1:** Preclinical data on opioid-induced gut dysbiosis and related effects on pain perception.

Study	Opioid RoA Dosage	Treatment Duration	Findings on Dysbiosis	Findings on Analgesia
Lee et al. 2018 [29]	i.p. morphine 10, 20, 30, 40 mg/kg BID vs. s.c. morphine (implanted pellet) 25 mg	4 days	Both intermittent or sustained morphine led to gut bacterial changes. Intermittent morphine increased relative abundance of *Ruminococcus* spp. and decreased *Lactobacillus* spp. Sustained morphine increased relative abundances in *Clostridium* spp. and the family *Rikenellaceae*.	Both intermittent and sustained morphine treatment regimens resulted in morphine tolerance. Intermittent, but not sustained, morphine altered microglial morphology, and hyperalgesia.
Banerjee et al. 2016 [31]	s.c. morphine (implanted pellets) 25 mg	24–48 h	Morphine induced significant gut microbial dysbiosis: expansion of gram-positive Firmicutes phylum and reduction of phylum Bacteroidetes (reduced Bacteroidetes/Firmicutes ratio). Elevated levels of IL17 were observed after morphine treatment. Gut dysbiosis was reversed by fecal transplantation. Morphine disrupted cholesterol/bile acid metabolism.	N.A.
Kang et al. 2017 [30]	i.p. morphine 10 mg/kg (acute) vs. s.c. morphine (implanted pellets) 75 mg (chronic)	5 days (after 5 days of ABX pretreatment)	Chronic, but not acute, administration of morphine, altered gut permeability, enhanced bacterial translocation, and increased IL-1beta. All these effects were prevented by ABX treatment.	Chronic morphine exposure resulted in antinociceptive tolerance (in tail immersion and acetic acid stretch assay). ABX reduced gut bacteria and prevented antinociceptive tolerance.
Wang et al. 2018 [39]	s.c. morphine 25 mg vs. s.c. naltrexone 30 mg vs. s.c. morphine and naltrexone (implanted pellets)	1–6 days +6 days post-treatment	At day 1, the microbiome from the morphine-treated group clustered distinctly from all other groups; bacterial translocation into the MLN was observed; this trend was maintained as long as day 6. Naltrexone antagonized these effects. At day 3, naltrexone-treated animals clustered distinctly from placebo (possible role for endogenous opioids in the basal host microbial profile).	Infection with *E. faecalis* augmented morphine induced analgesic tolerance (in tail flick test).
Abu et al. 2022 [40]	i.p. hydromorphone 10 mg/kg OD	3 days G11–G13 -beginning of GI development in the growing fetus -(G0 gestation day 0)	Brief hydromorphone exposure during pregnancy induced changes in both maternal and neonatal microbioma. In dams, Gram-negative bacteria increased and Gram-positive bacteria decreased. This trend was inverted in POE neonates.	N.A.
Abu et al. 2023 [41]	s.c. hydromorphone 0.5–3.5 mg/kg BID (0.75 mg/kg dose increments every 3 days) Followed by i.p. methadone 10 mg/kg BID	14 days pre-mating, then rotated to methadone	Methadone-exposed dams showed enriched aerobic, biofilm forming bacteria, and Gram-negative bacteria relative to control, and decreased relative abundance of Gram-positive bacteria. Dysbiosis of dams correlated with dysbiosis in POE neonates.	POE (methadone) increased sensitivity to thermal and mechanical pain. Supplementation with probiotics in dams altered neonatal gut microbiome and rescued hypersensitivity to thermal and mechanical pain.
Zhang et al. 2019 [34]	s.c. morphine 5–40 mg/kg BID Escalating doses	8 days	Chronic morphine induced gut dysbiosis: reduction in Actinobacteria and Firmicutes, Bifidobacteriaceae and Lactobacillaceae families, and Bifidobacterium and Lactobacillus gena; significant bacterial translocation was observed. Morphine initiated local gut inflammation through TLR2 and TLR4 activation (reversed in ABX mice): increased levels of proinflammatory cytokines IL6, IL1 beta, and TNF alfa.	Probiotic pretreatment attenuated morphine tolerance and prevented morphine-induced gut microbiota alterations.

RoA route of administration; i.p. intraperitoneal; BID twice-daily; s.c. subcutaneous; OD once daily; GI gastrointestinal; N.A. Not available; POE prenatal opioid exposure; ABX broad-spectrum antibiotics; MLN mesenteric lymph nodes; TLR tool-like receptors; TNF tumor necrosis factor.

## References

[1] Fallon M., Giusti R., Aielli F., Hoskin P., Rolke R., Sharma M., Ripamonti C.I. (2018). ESMO Guidelines Committee. Management of cancer pain in adult patients: ESMO Clinical Practice Guidelines. Ann. Oncol..

[2] Mercadante S., Coluzzi F. (2021). Factors Influencing Pain Expression in Patients with Cancer: An Expert Opinion. Pain. Ther..

[3] Alvaro D., Coluzzi F., Gianni W., Lugoboni F., Marinangeli F., Massazza G., Pinto C., Varrassi G. (2022). Opioid-Induced Constipation in Real-World Practice: A Physician Survey, 1 Year Later. Pain. Ther..

[4] Coluzzi F., Scerpa M.S., Pergolizzi J. (2020). Naldemedine: A New Option for OIBD. J. Pain. Res..

[5] Agulló L., Muriel J., Margarit C., Escorial M., Garcia D., Herrero M.J., Hervás D., Sandoval J., Peiró A.M. (2023). Sex Differences in Opioid Response Linked to OPRM1 and COMT genes DNA Methylation/Genotypes Changes in Patients with Chronic Pain. J. Clin. Med..

[6] Wilder-Smith C.H., Bettiga A. (1997). The analgesic tramadol has minimal effect on gastrointestinal motor function. Br. J. Clin. Pharmacol..

[7] Mark E.B., Nedergaard R.B., Hansen T.M., Nissen T.D., Frøkjaer J.B., Scott S.M., Krogh K., Drewes A.M. (2021). Tapentadol results in less deterioration of gastrointestinal function and symptoms than standard opioid therapy in healthy male volunteers. Neurogastroenterol. Motil..

[8] Alvaro D., Caraceni A.T., Coluzzi F., Gianni W., Lugoboni F., Marinangeli F., Massazza G., Pinto C., Varrassi G. (2020). What to Do and What Not to Do in the Management of Opioid-Induced Constipation: A Choosing Wisely Report. Pain. Ther..

[9] Coluzzi F., Alvaro D., Caraceni A.T., Gianni W., Marinangeli F., Massazza G., Pinto C., Varrassi G., Lugoboni F. (2021). Common Clinical Practice for Opioid-Induced Constipation: A Physician Survey. J. Pain. Res..

[10] Essmat N., Karádi D.Á., Zádor F., Király K., Fürst S., Al-Khrasani M. (2023). Insights into the Current and Possible Future Use of Opioid Antagonists in Relation to Opioid-Induced Constipation and Dysbiosis. Molecules.

[11] Coluzzi F., Scerpa M.S., Rocco M., Fornasari D. (2022). The Impact of P-Glycoprotein on Opioid Analgesics: What’s the Real Meaning in Pain Management and Palliative Care?. Int. J. Mol. Sci..

[12] Wang H., Luo J., Chen X., Hu H., Li S., Zhang Y., Shi C. (2022). Clinical Observation of the Effects of Oral Opioid on Inflammatory Cytokines and Gut Microbiota in Patients with Moderate to Severe Cancer Pain: A Retrospective Cohort Study. Pain. Ther..

[13] Zhang J., Yang J., Yang C., Chen T., Wang Z., Li J., Qin F., Deng Q., Zhang X. (2020). Sensitivity to Morphine Reward Associates With Gut Dysbiosis in Rats With Morphine-Induced Conditioned Place Preference. Front. Psychiatry.

[14] Sorboni S.G., Moghaddam H.S., Jafarzadeh-Esfehani R., Soleimanpour S. (2022). A Comprehensive Review on the Role of the Gut Microbiome in Human Neurological Disorders. Clin. Microbiol. Rev..

[15] Gebrayel P., Nicco C., Al Khodor S., Bilinski J., Caselli E., Comelli E.M., Egert M., Giaroni C., Karpinski T.M., Loniewski I. (2022). Microbiota medicine: Towards clinical revolution. J. Transl. Med..

[16] Berg G., Rybakova D., Fischer D., Cernava T., Vergès M.C., Charles T., Chen X., Cocolin L., Eversole K., Corral G.H. (2020). Microbiome definition re-visited: Old concepts and new challenges. Microbiome.

[17] Ibal J.C., Park Y.J., Park M.K., Lee J., Kim M.C., Shin J.H. (2022). Review of the Current State of Freely Accessible Web Tools for the Analysis of 16S rRNA Sequencing of the Gut Microbiome. Int. J. Mol. Sci..

[18] Coelho G.D.P., Ayres L.F.A., Barreto D.S., Henriques B.D., Prado M.R.M.C., Passos C.M.D. (2021). Acquisition of microbiota according to the type of birth: An integrative review. Rev. Lat. Am. Enferm..

[19] Gritz E.C., Bhandari V. (2015). The human neonatal gut microbiome: A brief review. Front. Pediatr..

[20] Hasan N., Yang H. (2019). Factors affecting the composition of the gut microbiota, and its modulation. PeerJ.

[21] Chang C., Yuan X., Zhang X., Chen X., Li K. (2022). Gastrointestinal Microbiome and Multiple Health Outcomes: Umbrella Review. Nutrients.

[22] Bull M.J., Plummer N.T. (2014). Part 1: The Human Gut Microbiome in Health and Disease. Integr. Med..

[23] Fan Y., Pedersen O. (2021). Gut microbiota in human metabolic health and disease. Nat. Rev. Microbiol..

[24] Zheng D., Liwinski T., Elinav E. (2020). Interaction between microbiota and immunity in health and disease. Cell Res..

[25] Carabotti M., Scirocco A., Maselli M.A., Severi C. (2015). The gut-brain axis: Interactions between enteric microbiota, central and enteric nervous systems. Ann. Gastroenterol..

[26] York A. (2021). Avoiding the pitfalls in microbiota studies. Nat. Rev. Microbiol..

[27] Cullen C.M., Aneja K.K., Beyhan S., Cho C.E., Woloszynek S., Convertino M., McCoy S.J., Zhang Y., Anderson M.Z., Alvarez-Ponce D. (2020). Emerging Priorities for Microbiome Research. Front. Microbiol..

[28] Wang H.X., Wang Y.P. (2016). Gut Microbiota-brain Axis. Chin. Med. J..

[29] Lee K., Vuong H.E., Nusbaum D.J., Hsiao E.Y., Evans C.J., Taylor A.M.W. (2018). The gut microbiota mediates reward and sensory responses associated with regimen-selective morphine dependence. Neuropsychopharmacology.

[30] Kang M., Mischel R.A., Bhave S., Komla E., Cho A., Huang C., Dewey W.L., Akbarali H.I. (2017). The effect of gut microbiome on tolerance to morphine mediated antinociception in mice. Sci. Rep..

[31] Banerjee S., Sindberg G., Wang F., Meng J., Sharma U., Zhang L., Dauer P., Chen C., Dalluge J., Johnson T. (2016). Opioid-induced gut microbial disruption and bile dysregulation leads to gut barrier compromise and sustained systemic inflammation. Mucosal Immunol..

[32] Mariat D., Firmesse O., Levenez F., Guimarăes V., Sokol H., Doré J., Corthier G., Furet J.P. (2009). The Firmicutes/Bacteroidetes ratio of the human microbiota changes with age. BMC Microbiol..

[33] Muchhala K.H., Kallurkar P.S., Kang M., Koseli E., Poklis J.L., Xu Q., Dewey W.L., Fettweis J.M., Jimenez N.R., Akbarali H.I. (2024). The role of morphine- and fentanyl-induced impairment of intestinal epithelial antibacterial activity in dysbiosis and its impact on the microbiota-gut-brain axis. FASEB J..

[34] Zhang L., Meng J., Ban Y., Jalodia R., Chupikova I., Fernandez I., Brito N., Sharma U., Abreu M.T., Ramakrishnan S. (2019). Morphine tolerance is attenuated in germfree mice and reversed by probiotics, implicating the role of gut microbiome. Proc. Natl. Acad. Sci. USA.

[35] Fürst S., Zádori Z.S., Zádor F., Király K., Balogh M., László S.B., Hutka B., Mohammadzadeh A., Calabrese C., Galambos A.R. (2020). On the Role of Peripheral Sensory and Gut Mu Opioid Receptors: Peripheral Analgesia and Tolerance. Molecules.

[36] Meng J., Banerjee S., Li D., Sindberg G.M., Wang F., Ma J., Roy S. (2015). Opioid Exacerbation of Gram-positive sepsis, induced by Gut Microbial Modulation, is Rescued by IL-17A Neutralization. Sci. Rep..

[37] Sharma U., Olson R.K., Erhart F.N., Zhang L., Meng J., Segura B., Banerjee S., Sharma M., Saluja A.K., Ramakrishnan S. (2020). Prescription opioids induce gut dysbiosis and exacerbate colitis in a murine model of infammatory Bowel disease. J. Crohns Colitis.

[38] Jalodia R., Abu Y.F., Oppenheimer M.R., Herlihy B., Meng J., Chupikova I., Tao J., Ghosh N., Dutta R.K., Kolli U. (2022). Opioid Use, Gut Dysbiosis, Inflammation, and the Nervous System. J. Neuroimmune Pharmacol..

[39] Wang F., Meng J., Zhang L., Johnson T., Chen C., Roy S. (2018). Morphine induces changes in the gut microbiome and metabolome in a morphine dependence model. Sci. Rep..

[40] Abu Y., Tao J., Dutta R., Yan Y., Vitari N., Kolli U., Roy S. (2022). Brief Hydromorphone Exposure During Pregnancy Sufficient to Induce Maternal and Neonatal Microbial Dysbiosis. J. Neuroimmune Pharmacol..

[41] Abu Y.F., Singh S., Tao J., Chupikova I., Singh P., Meng J., Roy S. (2023). Opioid-induced dysbiosis of maternal gut microbiota during gestation alters offspring gut microbiota and pain sensitivity. Gut Microbes.

[42] Nagpal R., Wang S., Solberg Woods L.C., Seshie O., Chung S.T., Shively C.A., Register T.C., Craft S., McClain D.A., Yadav H. (2018). Comparative Microbiome Signatures and Short-Chain Fatty Acids in Mouse, Rat, Non-human Primate, and Human Feces. Front. Microbiol..

[43] Sindberg G.M., Callen S.E., Banerjee S., Meng J., Hale V.L., Hegde R., Cheney P.D., Villinger F., Roy S., Buch S. (2019). Morphine Potentiates Dysbiotic Microbial and Metabolic Shifts in Acute SIV Infection. J. Neuroimmune Pharmacol..

[44] Shreiner A.B., Kao J.Y., Young V.B. (2015). The gut microbiome in health and in disease. Curr. Opin. Gastroenterol..

[45] Singh R., Zogg H., Ghoshal U.C., Ro S. (2022). Current Treatment Options and Therapeutic Insights for Gastrointestinal Dysmotility and Functional Gastrointestinal Disorders. Front. Pharmacol..

[46] Acharya C., Betrapally N.S., Gillevet P.M., Sterling R.K., Akbarali H., White M.B., Ganapathy D., Fagan A., Sikaroodi M., Bajaj J.S. (2017). Chronic opioid use is associated with altered gut microbiota and predicts readmissions in patients with cirrhosis. Aliment. Pharmacol. Ther..

[47] Barengolts E., Green S.J., Eisenberg Y., Akbar A., Reddivari B., Layden B.T., Dugas L., Chlipala G. (2018). Gut microbiota varies by opioid use, circulating leptin and oxytocin in African American men with diabetes and high burden of chronic disease. PLoS ONE.

[48] Mayer E.A., Tillisch K., Gupta A. (2015). Gut/brain axis and the microbiota. J. Clin. Investig..

[49] Zhao L., Xiong Q., Stary C.M., Mahgoub O.K., Ye Y., Gu L., Xiong X., Zhu S. (2018). Bidirectional gut-brain-microbiota axis as a potential link between inflammatory bowel disease and ischemic stroke. J. Neuroinflammation.

[50] Strandwitz P. (2018). Neurotransmitter modulation by the gut microbiota. Brain Res..

[51] Louwies T., Johnson A.C., Orock A., Yuan T., Greenwood-Van Meerveld B. (2020). The microbiota-gut-brain axis: An emerging role for the epigenome. Exp. Biol. Med..

[52] Hofford R.S., Mervosh N.L., Euston T.J., Meckel K.R., Orr A.T., Kiraly D.D. (2021). Alterations in microbiome composition and metabolic byproducts drive behavioral and transcriptional responses to morphine. Neuropsychopharmacology.

[53] Peirce J.M., Alviña K. (2019). The role of inflammation and the gut microbiome in depression and anxiety. J. Neurosci. Res..

[54] Osokine I., Erlebacher A. (2017). Inflammation and Autism: From Maternal Gut to Fetal Brain. Trends Mol. Med..

[55] García-Cabrerizo R., Carbia C., ORiordan K.J., Schellekens H., Cryan J.F. (2021). Microbiota-gut-brain axis as a regulator of reward processes. J. Neurochem..

[56] Rueda-Ruzafa L., Cruz F., Cardona D., Hone A.J., Molina-Torres G., Sánchez-Labraca N., Roman P. (2020). Opioid system influences gut-brain axis: Dysbiosis and related alterations. Pharmacol. Res..

[57] Sauma S., Casaccia P. (2020). Gut-brain communication in demyelinating disorders. Curr. Opin. Neurobiol..

[58] Koutzoumis D.N., Vergara M., Pino J., Buddendorff J., Khoshbouei H., Mandel R.J., Torres G.E. (2020). Alterations of the gut microbiota with antibiotics protects dopamine neuron loss and improve motor deficits in a pharmacological rodent model of Parkinson’s disease. Exp. Neurol..

[59] Zhuang Z.Q., Shen L.L., Li W.W., Fu X., Zeng F., Gui L., Lü Y., Cai M., Zhu C., Tan Y.L. (2018). Gut Microbiota is Altered in Patients with Alzheimer’s Disease. J. Alzheimers Dis..

[60] Stanislawski M.A., Stamper C.E., Stearns-Yoder K.A., Hoisington A.J., Brostow D.P., Forster J.E., Postolache T.T., Lowry C.A., Brenner L.A. (2021). Characterization of the gut microbiota among Veterans with unique military-related exposures and high prevalence of chronic health conditions: A United States-Veteran Microbiome Project (US-VMP) study. Brain Behav. Immun. Health.

[61] Lucerne K.E., Kiraly D.D. (2021). The role of gut-immune-brain signaling in substance use disorders. Int. Rev. Neurobiol..

[62] Taboun Z.S., Sadeghi J. (2023). The bidirectional relationship between opioids and the gut microbiome: Implications for opioid tolerance and clinical interventions. Int. Immunopharmacol..

[63] Chow A.K., Gulbransen B.D. (2017). Potential roles of enteric glia in bridging neuroimmune communication in the gut. Am. J. Physiol. Gastrointest. Liver Physiol..

[64] Bhave S., Gade A., Kang M., Hauser K.F., Dewey W.L., Akbarali H.I. (2017). Connexin-purinergic signaling in enteric glia mediates the prolonged effect of morphine on constipation. Faseb J..

[65] Pinho-Ribeiro F.A., Verri W.A., Chiu I.M. (2017). Nociceptor Sensory Neuron-Immune Interactions in Pain and Inflammation. Trends Immunol..

[66] Ji R.R., Xu Z.Z., Gao Y.J. (2014). Emerging targets in neuroinflammation-driven chronic pain. Nat. Rev. Drug Discov..

[67] Corder G., Tawfik V.L., Wang D., Sypek E.I., Low S.A., Dickinson J.R., Sotoudeh C., Clark J.D., Barres B.A., Bohlen C.J. (2017). Loss of mu opioid receptor signaling in nociceptors, but not microglia, abrogates morphine tolerance without disrupting analgesia. Nat. Med..

[68] Miller R.E., Ishihara S., Tran P.B., Golub S.B., Last K., Miller R.J., Fosang A.J., Malfait A.M. (2018). An aggrecan fragment drives osteoarthritis pain through Toll-like receptor 2. JCI Insight.

[69] Diogenes A., Ferraz C.C., Akopian A.N., Henry M.A., Hargreaves K.M. (2011). LPS sensitizes TRPV1 via activation of TLR4 in trigeminal sensory neurons. J. Dent. Res..

[70] Vinolo M.A., Rodrigues H.G., Nachbar R.T., Curi R. (2011). Regulation of inflammation by short chain fatty acids. Nutrients.

[71] Kukkar A., Singh N., Jaggi A.S. (2014). Attenuation of neuropathic pain by sodium butyrate in an experimental model of chronic constriction injury in rats. J. Formos. Med. Assoc..

[72] Banasiewicz T., Krokowicz Ł., Stojcev Z., Kaczmarek B.F., Kaczmarek E., Maik J., Marciniak R., Krokowicz P., Walkowiak J., Drews M. (2013). Microencapsulated sodium butyrate reduces the frequency of abdominal pain in patients with irritable bowel syndrome. Color. Dis..

[73] Erny D., Hrabě de Angelis A.L., Jaitin D., Wieghofer P., Staszewski O., David E., Keren-Shaul H., Mahlakoiv T., Jakobshagen K., Buch T. (2015). Host microbiota constantly control maturation and function of microglia in the CNS. Nat. Neurosci..

[74] Latremoliere A., Woolf C.J. (2009). Central sensitization: A generator of pain hypersensitivity by central neural plasticity. J. Pain..

[75] Tsuda M. (2018). Modulation of Pain and Itch by Spinal Glia. Neurosci. Bull..

[76] Ritz N.L., Draper L.A., Bastiaanssen T.F.S., Turkington C.J.R., Peterson V.L., van de Wouw M., Vlckova K., Fülling C., Guzzetta K.E., Burokas A. (2024). The gut virome is associated with stress-induced changes in behaviour and immune responses in mice. Nat. Microbiol..

[77] Skaper S.D., Facci L., Giusti P. (2013). Glia and Mast Cells as Targets for Palmitoylethanolamide, an Anti-inflammatory and Neuroprotective Lipid Mediator. Mol. Neurobiol..

[78] Di Cesare Mannelli L., Corti F., Micheli L., Zanardelli M., Ghelardini C. (2015). Delay of Morphine Tolerance by Palmitoylethanolamide. Biomed. Res. Int..

[79] Di Cesare Mannelli L., Micheli L., Lucarini E., Ghelardini C. (2018). Ultramicronized N-Palmitoylethanolamine Supplementation for Long-Lasting, Low-Dosed Morphine Antinociception. Front. Pharmacol..

[80] Micheli L., Lucarini E., Toti A., Ferrara V., Ciampi C., Parisio C., Bartolucci G., Di Cesare Mannelli L., Ghelardini C. (2022). Effects of Ultramicronized N-Palmitoylethanolamine Supplementation on Tramadol and Oxycodone Analgesia and Tolerance Prevention. Pharmaceutics.

[81] Cristiano C., Pirozzi C., Coretti L., Cavaliere G., Lama A., Russo R., Lembo F., Pina Mollica M., Meli R., Calignano A. (2018). Palmitoylethanolamide counteracts autistic-like behaviours in BTBR T+tf/J mice: Contribution of central and peripheral mechanisms. Brain Behav. Immun..

[82] Guida F., Boccella S., Belardo C., Iannotta M., Piscitelli F., De Filippis F., Paino S., Ricciardi F., Siniscalco D., Marabese I. (2020). Altered gut microbiota and endocannabinoid system tone in vitamin D deficiency-mediated chronic pain. Brain Behav. Immune.

[83] Guida F., Rocco M., Luongo L., Persiani P., Vulpiani M.C., Nusca S.M., Maione S., Coluzzi F. (2022). Targeting Neuroinflammation in Osteoarthritis with Intra-Articular Adelmidrol. Biomolecules.

[84] Del Re A., Palenca I., Seguella L., Pesce M., Corpetti C., Steardo L., Rurgo S., Sarnelli G., Esposito G. (2022). Oral Adelmidrol Administration Up-Regulates Palmitoylethanolamide Production in Mice Colon and Duodenum through a PPAR-γ Independent Action. Metabolites.

[85] Berridge K.C., Robinson T.E. (2003). Parsing reward. Trends Neurosci..

[86] Volkow N.D., Morales M. (2015). The Brain on Drugs: From Reward to Addiction. Cell.

[87] Senchukova M.A. (2023). Microbiota of the gastrointestinal tract: Friend or foe?. World J. Gastroenterol..

[88] Cryan J.F., O’Riordan K.J., Sandhu K., Peterson V., Dinan T.G. (2020). The gut microbiome in neurological disorders. Lancet Neurol..

[89] Kennedy P.J., Cryan J.F., Dinan T.G., Clarke G. (2017). Kynurenine pathway metabolism and the microbiota-gut-brain axis. Neuropharmacology.

[90] Joseph J., Depp C., Shih P.B., Cadenhead K.S., Schmid-Schönbein G. (2017). Modified Mediterranean Diet for Enrichment of Short Chain Fatty Acids: Potential Adjunctive Therapeutic to Target Immune and Metabolic Dysfunction in Schizophrenia?. Front. Neurosci..

[91] El-Ansary A.K., Bacha A., Ben K.M. (2012). Etiology of autistic features: The persisting neurotoxic effects of propionic acid. J. Neuroinflammation.

[92] Byrne C.S., Chambers E.S., Morrison D.J., Frost G. (2015). The role of short chain fatty acids in appetite regulation and energy homeostasis. Int. J. Obes..

[93] Shishov V.A., Kirovskaia T.A., Kudrin V.S., Oleskin A.V. (2009). Amine neuromediators, their precursors, and oxidation products in the culture of Escherichia coli K-12. Prikl. Biokhim Mikrobiol..

[94] Patterson E., Ryan P.M., Wiley N., Carafa I., Sherwin E., Moloney G., Franciosi E., Mandal R., Wishart D.S., Tuohy K. (2019). Gamma-aminobutyric acid-producing lactobacilli positively affect metabolism and depressive-like behaviour in a mouse model of metabolic syndrome. Sci. Rep..

[95] Al-Ghezi Z.Z., Busbee P.B., Alghetaa H., Nagarkatti P.S., Nagarkatti M. (2019). Combination of cannabinoids, delta-9-tetrahydrocannabinol (THC) and cannabidiol (CBD), mitigates experimental autoimmune encephalomyelitis (EAE) by altering the gut microbiome. Brain Behav. Immun..

[96] Koob G.F., Buck C.L., Cohen A., Edwards S., Park P.E., Schlosburg J.E., Schmeichel B., Vendruscolo L.F., Wade C.L., Whitfield T.W. (2014). Addiction as a stress surfeit disorder. Neuropharmacology.

[97] Rea K., Dinan T.G., Cryan J.F. (2016). The microbiome: A key regulator of stress and neuroinflammation. Neurobiol. Stress..

[98] Thomas K.R., Watt J., Wu C.M.J., Akinrinoye A., Amjad S., Colvin L., Cowe R., Duncan S.H., Russell W.R., Forget P. (2022). Pain and Opioid-Induced Gut Microbial Dysbiosis. Biomedicines.

[99] Qin C., Hu J., Wan Y., Cai M., Wang Z., Peng Z., Liao Y., Li D., Yao P., Liu L. (2021). Narrative review on potential role of gut microbiota in certain substance addiction. Prog. Neuropsychopharmacol. Biol. Psychiatry.

[100] Koustas E., Trifylli E.M., Sarantis P., Papadopoulos N., Aloizos G., Tsagarakis A., Damaskos C., Garmpis N., Garmpi A., Papavassiliou A.G. (2022). Implication of gut microbiome in immunotherapy for colorectal cancer. World J. Gastrointest. Oncol..

[101] Li W., Deng Y., Chu Q., Zhang P. (2019). Gut microbiome and cancer immunotherapy. Cancer Lett..

[102] Araji G., Maamari J., Ahmad F.A., Zareef R., Chaftari P., Yeung S.J. (2021). The Emerging Role of the Gut Microbiome in the Cancer Response to Immune Checkpoint Inhibitors: A Narrative Review. J. Immunother. Precis. Oncol..

[103] Verschueren M.V., van der Welle C.M.C., Tonn M., Schramel F.M.N.H., Peters B.J.M., van de Garde E.M.W. (2021). The association between gut microbiome affecting concomitant medication and the effectiveness of immunotherapy in patients with stage IV NSCLC. Sci. Rep..

[104] Gaucher L., Adda L., Séjourné A., Joachim C., Guillaume C., Poulet C., Liabeuf S., Gras-Champel V., Masmoudi K., Houessinon A. (2021). Associations between dysbiosis-inducing drugs, overall survival and tumor response in patients treated with immune checkpoint inhibitors. Ther. Adv. Med. Oncol..

[105] Hong S., Lee J.H., Heo J.Y., Suh K.J., Kim S.H., Kim Y.J., Kim J.H. (2024). Impact of concurrent medications on clinical outcomes of cancer patients treated with immune checkpoint inhibitors: Analysis of Health Insurance Review and Assessment data. J. Cancer Res. Clin. Oncol..

[106] Prasetya R.A., Metselaar-Albers M., Engels F. (2021). Concomitant use of analgesics and immune checkpoint inhibitors in non-small cell lung cancer: A pharmacodynamics perspective. Eur. J. Pharmacol..

[107] Sun M., Chang C.L., Lu C.Y., Zhang J., Wu S.Y. (2022). Effect of opioids on cancer survival in patients with chronic pain: A propensity score-matched population-based cohort study. Br. J. Anaesth..

[108] Tomkovich S., Taylor A., King J., Colovas J., Bishop L., McBride K., Royzenblat S., Lesniak N.A., Bergin I.L., Schloss P.D. (2021). An Osmotic Laxative Renders Mice Susceptible to Prolonged Clostridioides difficile Colonization and Hinders Clearance. mSphere.

[109] Rao V.L., Micic D., Davis A.M. (2019). Medical Management of Opioid-Induced Constipation. JAMA.

[110] Varrassi G., Coluzzi F., Guardamagna V.A., Puntillo F., Sotgiu G., Vellucci R., Rational Use of Analgesics (RUA) Group (2021). Personalizing Cancer Pain Therapy: Insights from the Rational Use of Analgesics (RUA) Group. Pain. Ther..

[111] Pandey H., Tang D.W.T., Wong S.H., Lal D. (2023). Gut Microbiota in Colorectal Cancer: Biological Role and Therapeutic Opportunities. Cancers.

[112] Saab S., Suraweera D., Au J., Saab E.G., Alper T.S., Tong M.J. (2016). Probiotics are helpful in hepatic encephalopathy: A meta-analysis of randomized trials. Liver Int..

[113] Rousseaux C., Thuru X., Gelot A., Barnich N., Neut C., Dubuquoy L., Dubuquoy C., Merour E., Geboes K., Chamaillard M. (2007). *Lactobacillus acidophilus* modulates intestinal pain and induces opioid and cannabinoid receptors. Nat. Med..

[114] Yuan W., Xiao J., Liao H., Xie Z., Zhao Y., Li C., Zhou K., Song X.J. (2023). *Lactobacillus rhamnosus* GG and butyrate supplementation in rats with bone cancer reduces mechanical allodynia and increases expression of μ-opioid receptor in the spinal cord. Front. Mol. Neurosci..

[115] Zhao K., Yu L., Wang X., He Y., Lu B. (2018). Clostridium butyricum regulates visceral hypersensitivity of irritable bowel syndrome by inhibiting colonic mucous low grade inflammation through its action on NLRP6. Acta Biochim. Biophys. Sin..

[116] McKernan D.P., Fitzgerald P., Dinan T.G., Cryan J.F. (2010). The probiotic Bifidobacterium infantis 35624 displays visceral antinociceptive effects in the rat. Neurogastroenterol. Motil..

[117] Li Y.J., Dai C., Jiang M. (2019). Mechanisms of Probiotic VSL#3 in a Rat Model of Visceral Hypersensitivity Involves the Mast Cell-PAR2-TRPV1 Pathway. Dig. Dis. Sci..

[118] Kannampalli P., Pochiraju S., Chichlowski M., Berg B.M., Rudolph C., Bruckert M., Miranda A., Sengupta J.N. (2014). Probiotic Lactobacillus rhamnosus GG (LGG) and prebiotic prevent neonatal inflammation-induced visceral hypersensitivity in adult rats. Neurogastroenterol. Motil..

[119] Bi K., Lei Y., Kong D., Li Y., Fan X., Luo X., Yang J., Wang G., Li X., Xu Y. (2024). Progress in the study of intestinal microbiota involved in morphine tolerance. Heliyon.

[120] Ait-Belgnaoui A., Payard I., Rolland C., Harkat C., Braniste V., Théodorou V., Tompkins T.A. (2018). *Bifidobacterium longum* and *Lactobacillus helveticus* Synergistically Suppress Stress-related Visceral Hypersensitivity Through Hypothalamic-Pituitary-Adrenal Axis Modulation. J. Neurogastroenterol. Motil..

[121] Weizman Z., Abu-Abed J., Binsztok M. (2016). *Lactobacillus reuteri* DSM 17938 for the Management of Functional Abdominal Pain in Childhood: A Randomized, Double-Blind, Placebo-Controlled Trial. J. Pediatr..

[122] Spiller R., Pélerin F., Cayzeele Decherf A., Maudet C., Housez B., Cazaubiel M., Jüsten P. (2016). Randomized double blind placebo-controlled trial of Saccharomyces cerevisiae CNCM I-3856 in irritable bowel syndrome: Improvement in abdominal pain and bloating in those with predominant constipation. United Eur. Gastroenterol. J..

[123] Fernández Forné Á., García Anaya M.J., Segado Guillot S.J., Plaza Andrade I., de la Peña Fernández L., Lorca Ocón M.J., Lupiáñez Pérez Y., Queipo-Ortuño M.I., Gómez-Millán J. (2023). Influence of the microbiome on radiotherapy-induced oral mucositis and its management: A comprehensive review. Oral. Oncol..

[124] Cuozzo M., Castelli V., Avagliano C., Cimini A., d’Angelo M., Cristiano C., Russo R. (2021). Effects of Chronic Oral Probiotic Treatment in Paclitaxel-Induced Neuropathic Pain. Biomedicines.

[125] Lang T., Zhu R., Zhu X., Yan W., Li Y., Zhai Y., Wu T., Huang X., Yin Q., Li Y. (2023). Combining gut microbiota modulation and chemotherapy by capecitabine-loaded prebiotic nanoparticle improves colorectal cancer therapy. Nat. Commun..

[126] Ting N.L., Lau H.C., Yu J. (2022). Cancer pharmacomicrobiomics: Targeting microbiota to optimise cancer therapy outcomes. Gut.

[127] Guo R., Chen L.H., Xing C., Liu T. (2019). Pain regulation by gut microbiota: Molecular mechanisms and therapeutic potential. Br. J. Anaesth..

[128] Vulevic J., Tzortzis G., Juric A., Gibson G.R. (2018). Effect of a prebiotic galactooligosaccharide mixture (B-GOS^®^) on gastrointestinal symptoms in adults selected from a general population who suffer with bloating, abdominal pain, or flatulence. Neurogastroenterol. Motil..

[129] Cammarota G., Ianiro G., Cianci R., Bibbò S., Gasbarrini A., Currò D. (2015). The involvement of gut microbiota in inflammatory bowel disease pathogenesis: Potential for therapy. Pharmacol. Ther..

[130] Thurm T., Ablin J., Buskila D., Maharshak N. (2017). Faecal microbiota transplantation for fibromyalgia: A case report and review of the literature. Open J. Gastroenterol..

[131] Mischel R.A., Dewey W.L., Akbarali H.I. (2018). Tolerance to Morphine-Induced Inhibition of TTX-R Sodium Channels in Dorsal Root Ganglia Neurons Is Modulated by Gut-Derived Mediators. iScience.

